# Are mice good models for human neuromuscular disease? Comparing muscle excursions in walking between mice and humans

**DOI:** 10.1186/s13395-017-0143-9

**Published:** 2017-11-16

**Authors:** Xiao Hu, James P. Charles, Turgay Akay, John R. Hutchinson, Silvia S. Blemker

**Affiliations:** 1 Department of Biomedical Engineering, University of Virginia, 415 Lane Road, Charlottesville, VA 22908 USA; 2 Department of Orthopaedic Surgery, University of Pittsburgh, Pittsburgh, PA 15213 USA; 3 Department of Medical Neuroscience, Dalhousie University, Halifax, NS B3H 4R2 Canada; 4 Comparative Biomedical Sciences, Royal Veterinary College, Hatfield, Hertfordshire, AL9 7TA UK; 5 Department of Orthopaedic Surgery, University of Virginia, Charlottesville, VA 22903 USA; 6 Department of Mechanical and Aerospace Engineering, University of Virginia, Charlottesville, VA 22903 USA

**Keywords:** Neuromuscular diseases, Duchenne muscular dystrophy, Musculoskeletal simulation, Muscle fiber excursion, Walking gait, Mice, Biomechanics

## Abstract

**Background:**

The mouse is one of the most widely used animal models to study neuromuscular diseases and test new therapeutic strategies. However, findings from successful pre-clinical studies using mouse models frequently fail to translate to humans due to various factors. Differences in muscle function between the two species could be crucial but often have been overlooked. The purpose of this study was to evaluate and compare muscle excursions in walking between mice and humans.

**Methods:**

Recently published musculoskeletal models of the mouse hindlimb and human lower limb were used to simulate muscle-tendon dynamics during mouse and human walking, a key daily activity. Muscle fiber length changes (fiber excursions) of 25 muscle homologs in the two species were calculated from these simulations and then compared. To understand potential causes of differences in fiber excursions in walking, joint excursions and muscle moment arms were also compared across one gait cycle.

**Results:**

Most muscles (19 out of 25 muscles) of the mouse hindlimb had much smaller fiber excursions as compared to human lower limb muscles during walking. For these muscles, fiber excursions in mice were only 48 ± 19% of those in humans. The differences in fiber excursion between the two species were primarily due to the reduced joint excursions and smaller muscle moment arms in mice as compared to humans.

**Conclusions:**

Since progressive neuromuscular diseases, such as Duchenne muscular dystrophy, are known to be accelerated by damage accumulated from active muscle lengthening, these results suggest that biomechanical differences in muscle function during walking between mice and humans may impede the translations of knowledge gained from mouse models to humans. This knowledge would add a fresh perspective on how pre-clinical studies on mice might be better designed to improve translation to human clinical trials.

**Electronic supplementary material:**

The online version of this article (10.1186/s13395-017-0143-9) contains supplementary material, which is available to authorized users.

## Background

The mouse is one of the most widely used animal models to study neuromuscular diseases and test new treatments [1]. Numerous mouse models of a wide variety of human neuromuscular diseases have been developed, leading to valuable insights about the underlying pathophysiology of diseases and the identification of new potential therapeutic strategies [2–5]. Although mouse models play a crucial role for developing new therapies, treatments that succeed in pre-clinical studies using mouse models often fail in human trials [6–8]. Understanding the obstacles that limit the translation of therapies from mouse to human will accelerate future discoveries of new therapies, minimize attrition of investigational drugs, and lower the associated costs of financial, material, and human resources.

The difficulties in the translation from mouse pre-clinical trials to successful human trials may arise from two major sources [7]. First, flaws and inconsistencies in experimental design, such as insufficient sample size, improper randomization and blinding, and inappropriate selection of readout parameters (among others), may hinder this translation [9, 10]. In an attempt to address these issues, guidelines for standardizing methodologically rigorous pre-clinical experimental studies on mouse models have been established [11–13]. Second, anatomical and physiological differences between mouse models and their human counterparts may prevent the models from accurately reflecting disease progression in humans. For example, there are notable differences in ontogeny, immunology, and pathology between mice and humans, as well as considerable differences in the biomechanics of musculoskeletal systems [1, 14, 15]. Although substantial effort has been devoted to understanding the differences in growth and pathophysiology between mice and humans, the implications of the biomechanical differences between two species often have been overlooked.

The biomechanical differences between mice and humans could lead to the different phenotype appearing in mouse models of neuromuscular diseases and the diminished utility of pre-clinical studies for predicting the efficacy of new therapeutic treatments in humans. For example, in the widely used mouse model (*mdx* mice) of Duchenne muscular dystrophy (DMD), a genetic, progressive degenerative disorder of muscles affecting 1 in 3500 male births [16], the limb muscles in the *mdx* mice have a much milder phenotype than those in humans [17]. Differences in muscle function during daily locomotion, such as walking and running, between mice and humans may contribute to this milder phenotype in mice. For example, if mouse muscles stretch less during movement than human muscles, the extent of stretch-induced damage to dystrophic muscles would also be diminished [18–20]. However, whether the relative muscle excursions of mice during daily locomotion are smaller than those of humans remains unknown. In addition, current standard protocols recommended by the TREAT-NMD Neuromuscular Network [21] and many pre-clinical studies [22–25] that aimed to test the efficacy of new treatments in mouse models exercise *mdx* mice at 12 m/min (0.2 m/s). Although the forced walking exercise at this speed tends to exacerbate muscle damage in *mdx* mice [23, 24], the treatment efficacy demonstrated from these pre-clinical studies has not translated well to humans [26–31]. It is unclear whether the muscle fiber excursions in mice during this type of exercise are comparable to those humans may experience. So far, although sarcomere lengths of mouse muscle fibers at fixed joint postures have been measured in vivo [32, 33], experimental studies alone have not been able to shed light on muscle fiber excursions in mouse limb muscles during daily locomotion. One underlying challenge is that due to their small body size, it remains almost impossible to experimentally measure dynamic in vivo muscle fiber excursions in mice even with the state-of-the-art techniques.

Musculoskeletal modeling and simulation provide a useful non-invasive means to examine muscle function, such as how muscle fibers change length during locomotion. In musculoskeletal models, joint kinematics, muscle lines of action from origin to insertion, and muscle architecture parameters (such as physiological cross-sectional area, optimal fiber length, and pennation angle) are estimated based on extensive experimental measurements [34]. Each individual muscle is modeled with one or multiple muscle-tendon units from origin to insertion, and its dynamic contractile capability is represented using Hill-type muscle models [35]. The three-dimensional (3D) musculoskeletal model of human lower limb developed by Delp et al. [34] has been widely used to study human locomotion and has evolved through the years to incorporate more accurate anatomic data from large-scale cadaver and imaging studies [36, 37]. Simulation studies using this model have evaluated the muscle fiber excursions in human walking and running at various speeds [38, 39]. Recently, a 3D musculoskeletal model of a mouse’s hindlimb [40] has been developed based on detailed anatomical measurements from microCT scanning, digital segmentation, and microdissection [41]. This model provides opportunities for simulation studies to examine the functions of mouse hindlimb muscles during locomotion, including the muscle fiber excursions, and to make comparisons with humans or other species.

The purpose of this study was to evaluate the muscle fiber excursions of mouse hindlimb muscles in walking and compare them to the excursions human lower limb muscles may experience in walking. Mice are non-cursorial quadrupeds [42] and therefore employ more flexed hip and knee postures during locomotion [43] relative to the more cursorial (i.e., straight-limbed/upright) humans [44]. We therefore hypothesize that the fiber excursions of mouse hindlimb muscles in walking are smaller than those of human lower limb muscles. To test this hypothesis, we used musculoskeletal models of the mouse hindlimb [40] and human lower limb [37] to run dynamic simulations of mice and humans walking at their most frequently used speeds in locomotion and to estimate fiber excursions of 25 muscle homologs in these two species. These results will shed new light on the potential implications of the biomechanical differences between mice and humans and could thereby lead to a fresh perspective on how pre-clinical studies on mice might be better designed to improve translation to human clinical trials. All the data and files used to create the simulations are freely available at https://simtk.org/projects/mice.

## Methods

### Musculoskeletal models

**Fig. 1:**
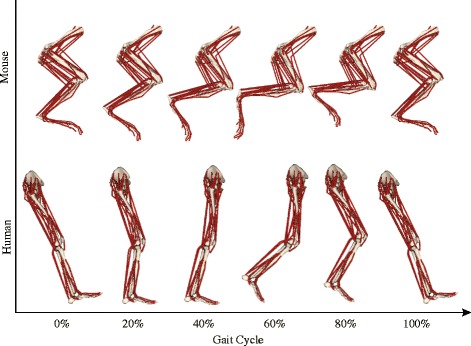
Musculoskeletal models (right side; lateral view) of the mouse hindlimb and human lower limb during one gait cycle. Each muscle was represented by one or multiple line-segment muscle-tendon units. The mechanical properties of each muscle-tendon unit were characterized by a Hill-type muscle model [35], which used separate elements to represent tendon and muscle fiber (Fig. 2)

Two state-of-the-art 3D musculoskeletal models of mouse hindlimb and human lower limb were used in this study to develop forward dynamic simulations of muscle-tendon dynamics during walking and estimate muscle fiber excursions (Fig. 1). The musculoskeletal model of a mouse hindlimb (https://simtk.org/projects/mousehindlimb) [40] includes 7 degrees of freedom to describe flexion/extension, adduction/abduction, and external/internal rotation of the hip joint; flexion/extension of the knee joint; and dorsiflexion/plantarflexion, eversion/inversion, and adduction/abduction of the ankle joint. A total of 44 muscle-tendon units were used to represent the 39 muscles of the mouse hindlimb and pelvis. Similarly, the musculoskeletal model of the human lower limbs (https://simtk.org/projects/full_body/) [37] includes 7 degrees of freedom for each leg to describe flexion/extension, adduction/abduction, and external/internal rotation of the hip joint; flexion/extension of the knee joint; dorsiflexion/plantarflexion and eversion/inversion of the ankle joint; and flexion/extension of the metatarsophalangeal joint. A total of 40 muscle-tendon units per leg were used to represent the 31 muscles of the human lower limb.

**Fig. 2:**
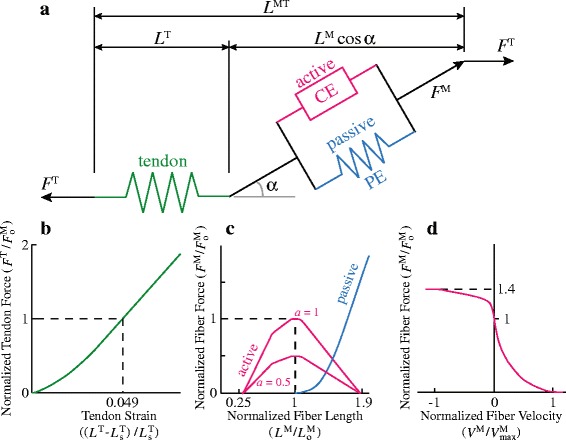
The contraction dynamics and force-generating capacity of each muscle-tendon unit was represented by a Hill-type muscle model [35]. (**a**) The total muscle-tendon length (*L* ^MT^) was a function of the geometric pose of the musculoskeletal models of mouse hindlimb and human lower limb. The muscle model computes muscle fiber length (*L* ^M^), muscle pennation angle (*α*), tendon length (*L* ^T^), muscle fiber force (*F* ^M^), and tendon force (*F* ^T^) based on *L* ^MT^, muscle activation (*a*), and the force equilibrium constraints between *F* ^M^ and *F* ^T^. (**b**) Tendon was modeled as a non-linear, passive series elastic element, whose mechanical property was defined by the tendon force-strain curve. In this curve, it was assumed that tendon strain (*ε* ^T^) is 4.9% when muscle fiber developed maximum isometric force (${F}_{\mathrm{o}}^{\mathrm{M}}$). Tendon strain was calculated from the muscle-specific tendon slack length (${L}_{\mathrm{s}}^{\mathrm{T}}$). (**c**) Muscle fiber was modeled as an active contractile element (CE) in parallel with a passive elastic element (PE). The active force-length curve was scaled by muscle-specific optimal fiber length (${L}_{\mathrm{o}}^{\mathrm{M}}$) and then used to compute active isometric fiber force based on *L* ^M^ and activation (*a*). The passive force-length curve was also scaled by ${L}_{\mathrm{o}}^{\mathrm{M}}$ and then used to compute passive fiber force based on *L* ^M^. (**d**) The active isometric fiber force was scaled based on fiber velocity (*V* ^M^) normalized by maximum shortening velocity (${V}_{\mathrm{max}}^{\mathrm{M}}$) of the muscle. Total muscle force was calculated as the sum of active and passive fiber force

In both musculoskeletal models, each muscle was approximated with one or more muscle-tendon units, depending on the size of attachment areas. Each muscle-tendon unit was represented as a massless series of line segments defining the path of a muscle from its origin to insertion [45]. A Hill-type muscle model [35] was used to characterize the contraction dynamics and force-generating capacity of each muscle-tendon unit. This muscle model used an active contractile element in parallel with a passive elastic element together to represent the muscle fiber and its active and passive force-generating capacity. The tendon of each muscle-tendon unit was represented using a non-linear, passive elastic element and was in series with the muscle fiber which had a pennation angle (*α*) (Fig. 2a). The mechanical properties of muscle fiber and tendon were defined using a set of generic fiber force-length, fiber force-velocity, and tendon force-strain curves (Fig. 2b–d). These curves were then scaled to characterize the unique architectural and mechanical properties of each muscle-tendon unit using five parameters (muscle maximum isometric force ${F}_{\mathrm{o}}^{\mathrm{M}}$, optimal fiber length ${L}_{\mathrm{o}}^{\mathrm{M}}$, maximum fiber shortening velocity ${V}_{\mathrm{max}}^{\mathrm{M}}$, pennation angle at optimal fiber length *α*_o_, and tendon slack length ${L}_{\mathrm{s}}^{\mathrm{T}}$). These parameters were derived from architectural data of each muscle gathered experimentally from mice and human cadavers via dissections and imaging techniques [37, 40, 41, 46], so the differences in muscle architecture in various muscles in mice and humans can be taken into account. In this model, activation (*a*) ranges from 0 to 1, representing passive to fully activated muscles, respectively. For muscle-tendon units in which the ratio of the optimal fiber length to the tendon slack length (${L}_{\mathrm{o}}^{\mathrm{M}}/{L}_{\mathrm{s}}^{\mathrm{T}}$) was larger than 2, tendons were assumed to be rigid considering the computational expenses and the physiological structure of muscles. This assumption only affected 10 muscles in mice and 3 in humans out of 25 muscles that were compared (Additional file 1: figure S1). These muscles were all upper leg (thigh) muscles that had no or very short external tendons [41, 47]. Therefore, tendon strain in these muscles should be negligible, and assuming a rigid tendon for these muscles allowed for significantly decreased simulation times [35] while barely affecting estimates of muscle fiber excursion (Additional file 1: figure S2). None of the lower leg (shank) muscles with measurable external tendons were affected. (see Additional file 1 for more explanation and which specific muscles were affected by the rigid tendon assumption.)

### Simulation of fiber length changes during walking

Simulations were produced by inputting joint kinematics previously published from two studies: 16 wild-type mice walking on a treadmill at the speed of 0.2 m/s [43] and 5 healthy human subjects walking on a treadmill at 1.25 m/s [39]. Because speed affects fiber excursions during locomotion [39], these two speeds were specifically chosen because they are comparable in the context of daily locomotion of two species. Previous studies have shown that the average spontaneous free walking speeds of mice and humans are about 0.2 [48, 49] and 1.25 m/s [44], respectively.

**Fig. 3:**
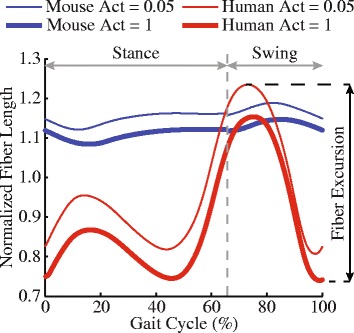
Simulated fiber length changes of the vastus lateralis muscle in dynamic models of mouse and human during one gait cycle. Fiber excursion in walking was defined as the difference between the maximum fiber length when activation (Act) was 0.05 and the minimum fiber length when activation was 1

Forward dynamic simulations of muscle-tendon dynamics during walking were developed using an open-source musculoskeletal simulation platform, OpenSim version 3.2 (https://simtk.org/projects/opensim) [50]. Experimentally measured joint kinematics of one gait cycle of each individual mouse and human was prescribed in the simulations. Because the mouse kinematic data from Akay et al. [43] were only available in the sagittal plane, including flexion/extension at the hip, knee, and ankle joints, kinematic data from humans were simplified to only include these degrees of freedom with all other degrees of freedom locked at default positions. To consider the influences of muscle activation on the possible ranges of fiber excursions, simulations were created with two activation cases (Fig. 3). In the first set of simulations, the activation levels for all the muscles were set to be a constant value of 1 (maximal activation). In the second set of simulations, the activation levels for all the muscles were set to be a constant value of 0.05 (minimum activation). The minimum activation was set at 0.05 to avoid numerical singularity in the muscle model when activation approaches 0 [35]. For both sets of simulations, the identical joint angle trajectories were prescribed based on the experimental data. Forward dynamic simulations were then run to calculate muscle fiber length (*L* ^M^) based on muscle-tendon length (*L* ^MT^), prescribed muscle activation (*a*), and the force equilibrium constraints between fiber force (*F* ^M^) and tendon force (*F* ^T^) that satisfied the prescribed motion [51]. Outputs of muscle fiber length were then analyzed to obtain fiber excursion (see Data analyses). All the simulations were developed using the generic models of the mouse hindlimb and human lower limbs and the specific joint kinematics that were measured from each individual mouse or human subject.

### Data analyses

Fiber length changes of each muscle-tendon unit during one gait cycle were estimated for the maximum and minimum activation cases separately and were then normalized to each muscle’s optimal fiber length. The muscle fiber excursions in walking were defined as the difference between the maximum fiber length when activation was 0.05 and the minimum fiber length when activation was 1 (Fig. 3). For a muscle approximated by multiple muscle-tendon units, fiber excursions of those muscle-tendon units were averaged to represent the mean fiber excursion of that whole muscle. Fiber excursions of 25 muscles that have homologs in the two species and are available both in musculoskeletal models of mice hindlimb and human lower limb were compared using a *t*-test with *p* values corrected by the Holm–Bonferroni method. The *p* value for significance was set at 0.05.

To understand potential causes of differences in fiber excursion in walking, we also compared the joint excursions and muscle moment arms between all mice and human subjects across one gait cycle. These analyses were performed because changes in length of a muscle-tendon unit are determined by: (*i*) the excursion of the joint it spans and (*ii*) its moment arm with regard to that joint [52]. Ranges of excursions for the hip, knee, and ankle joints were computed as the differences between the largest and the smallest joint angles in one gait cycle from the prescribed joint kinematics in the simulations. In addition, muscle flexion/extension moment arms at the hip, knee, and ankle in one gait cycle were calculated, averaged across one gait cycle, and then normalized by each muscle’s optimal fiber length. Similar *t*-tests with *p* value set at 0.05 were used to compare excursions of each joint and normalized muscle moment arms of each muscle (also corrected by Holm–Bonferroni method) between mice and human subjects.

**Fig. 4:**
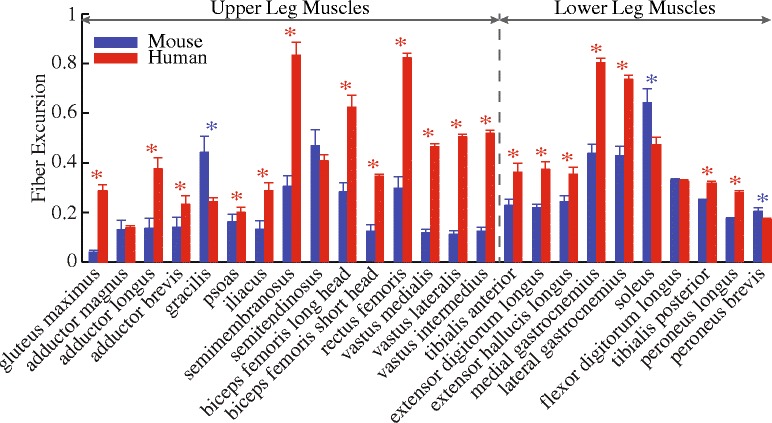
Comparison of the fiber excursions (mean and standard deviation) during walking between mice and humans. Blue stars indicate mice have larger excursions, while red stars indicate humans have larger excursions (*p* < 0.05)

To examine the potential impact of muscle fiber excursions in walking to the differential involvement in limb muscles in neuromuscular diseases, such as DMD, linear regression analyses were conducted to examine whether correlation exists between fiber excursions during gait and the fat fraction in patients with DMD, which is a metric of the extent of muscle degeneration. In the linear regression analyses, the fiber excursions were the average of excursions of individual muscle across five healthy human subjects (red bars in Fig. 4), and the fat fractions were acquired from a study by Wokke et al. [53], which quantified the fat fractions of lower limb muscles in 16 patients with DMD using quantitative magnetic resonance imaging (Fig. 2 in [53]). Separate linear regression analyses were conducted for upper leg and lower leg muscles (Fig. 4), respectively, because proximal upper leg muscles are generally more affected than the distal lower leg muscles in DMD [53, 54]. Four upper leg muscles (gluteus maximus, adductor brevis, psoas, and iliacus) of which fat fractions were not reported in [53] were not included in the analyses. Fiber excursions of peroneus longus and peroneus brevis were averaged in the analyses since the fat fraction was measured from the whole peroneus muscle in [53]. The level of significance was set at *p* < 0.05.

### Sensitivity analyses

Because of the intrinsic variability in physiological parameters of the muscle model, sensitivity analyses were conducted to evaluate the sensitivity of fiber excursions estimated from our simulations to two muscle-specific parameters: optimal fiber length${L}_{\mathrm{o}}^{\mathrm{M}}$ and tendon slack length ${L}_{\mathrm{s}}^{\mathrm{T}}$, as well as one parameter that defines normalized generic tendon force-strain curve: the tendon strain ${\varepsilon}_{\mathrm{o}}^{\mathrm{T}}$ when the normalized tendon force is one. Specifically,${L}_{\mathrm{o}}^{\mathrm{M}}$ and ${L}_{\mathrm{s}}^{\mathrm{T}}$ of each muscle-tendon unit were varied by its ± 1 standard deviation reported in [37, 41]. ${\varepsilon}_{\mathrm{o}}^{\mathrm{T}}$ is a parameter that defines the shape of the default generic force-strain curve of tendon, essentially the stiffness of the tendon. It has a default nominal value of 4.9% in the muscle model [35] and a plausible range of 2–9% [45]. Therefore, sensitivity analyses of ${\varepsilon}_{\mathrm{o}}^{\mathrm{T}}$ were conducted by setting its values at 2 and 9%, representing the stiffest and most compliant scenarios, respectively.

Sensitivity analyses were conducted separately for the mouse hindlimb model and human lower limb model. For each species, the averaged joint kinematics in one gait cycle across either all mice or all human subjects were prescribed. Dynamic simulations were first run with all three parameters at their default values to produce the nominal fiber excursions of each of the 25 muscles compared. Next, dynamic simulations were run with varying one parameter (${L}_{\mathrm{o}}^{\mathrm{M}}$or ${L}_{\mathrm{s}}^{\mathrm{T}}$ by ± 1 standard deviation, or ${\varepsilon}_{\mathrm{o}}^{\mathrm{T}}$ at 2 or 9%) while holding other two at their default values. The differences between the nominal fiber excursions and those excursions obtained with one of the three parameters varied were calculated and normalized by the optimal fiber length of each muscle. The sensitivity of fiber excursions to a given parameter was reported as the averaged differences across 25 muscles.

## Results

Most muscles of the mouse hindlimb had smaller fiber excursions during walking as compared to those of human lower limbs (Fig. 4). Out of the 25 muscles compared, 19 muscles had significantly smaller fiber excursions in mice as compared to humans. The fiber excursions of these muscles on average were $\left(0.25\pm 0.15\right)\ast {L}_{\mathrm{o}}^{\mathrm{M}}$ (mean ± standard deviation) smaller in mice than in humans. That is, the fiber excursions of these muscles in mice were only 48 ± 19% of those in humans. By contrast, only three muscles (gracilis, soleus, and peroneus brevis) had significantly larger fiber excursions in mice than in humans, and three muscles (adductor magnus, semitendinosus, and flexor digitorum longus) had comparable fiber excursions in the two species.

**Fig. 5:**
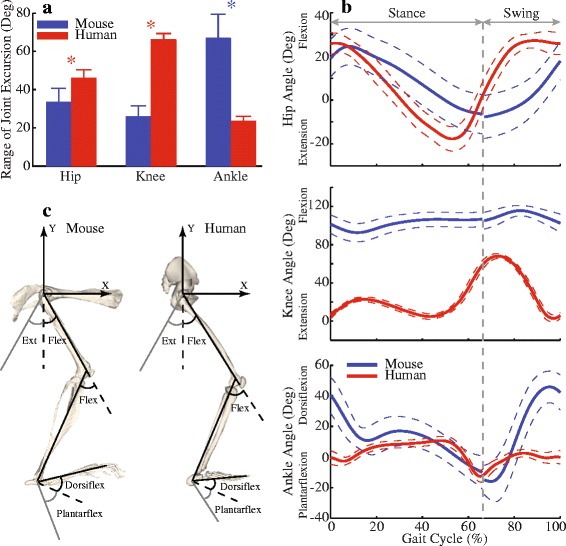
Comparison of the joint angles during one gait cycle between mice and humans. (**a**) Comparison of ranges of joint excursions (mean and standard deviation) at the hip, knee, and ankle joints between mice and humans. Blue stars indicate mice have larger ranges, while red stars indicate humans have larger ranges (*p* < 0.05). (**b**) Average of joint angles during one gait cycle (solid curves), from heel strike to heel strike. Dashed curves indicate ± standard deviation. Original gait data for mice started with toe-off. To be consistent with general gait data representation, the swing phase data from mice were manually moved to be after the stance phase. Vertical dashed line indicates toe-off at about 65% of a gait cycle for mice and humans. (**c**) Definition of the flexions and extensions at the hip, knee, and ankle joints. The flexion of the hip joint was defined relative to the coordinates of the pelvis (shown). *Flex* flexion, *Ext* extension, *Dorsiflex* dorsiflexion, *Plantarflex* plantarflexion

The joint excursions at the hip, knee, and ankle differed between mice and humans during walking (Fig. 5a). The differences were most pronounced at the knee and ankle joints. The knee joint excursion was 40.3° smaller in mice than in humans (Fig. 5b) because the knee joint of mice was kept at much more flexed joint angles (minimum knee flexion angle 90.5°) than that of humans (maximum knee flexion angle 68.3°). By contrast, the ankle joint excursion was 43.3° larger in mice than in humans, largely because mice dorsiflexed their ankles more than human subjects did, especially at the beginning of the stance phase and the end of the swing phase (Figs. 1 and 5b). While there was a statistical difference in the hip joint excursion between mice and humans, the difference was only 12.4° (Fig. 5a).

**Fig. 6:**
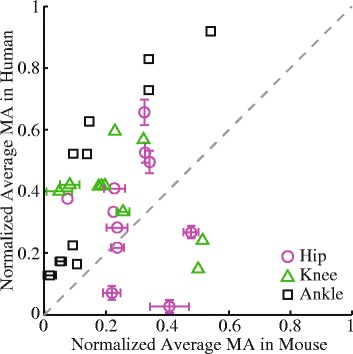
Comparison of average normalized moment arms (MA) of muscles crossing the hip, knee, and ankle joints in one gait cycle between mice and humans. Each symbol is one muscle. The dashed line is the unity line representing equal moment arms between mice and humans. Standard deviations that are smaller than the symbol are not shown

Almost all mouse hindlimb muscles had significantly smaller normalized average moment arms than human lower limb muscles during walking (Fig. 6). Seven out of 11 muscles crossing the hip joint, 8 out of 10 muscles crossing the knee joint, and all 10 muscles crossing the ankle joint had significantly smaller normalized flexion/extension moment arms in mice than in humans. For these muscles with smaller moment arms, the normalized moment arms in the mouse hindlimb were on average $\left(0.19\pm 0.10\right)\ast {L}_{\mathrm{o}}^{\mathrm{M}}$, $\left(0.26\pm 0.09\right)\ast {L}_{\mathrm{o}}^{\mathrm{M}}$ and $\left(0.30\pm 0.17\right)\ast {L}_{\mathrm{o}}^{\mathrm{M}}$ smaller than in the human lower limb for the hip, knee, and ankle joints, respectively. Only a few muscles had larger normalized moment arms during walking in mice than in humans. Adductor magnus and psoas had larger normalized moment arms at the hip joint. Gracilis and semitendinosus (both biarticular muscles) had larger normalized moment arms at the hip and the knee joints.

**Fig. 7:**
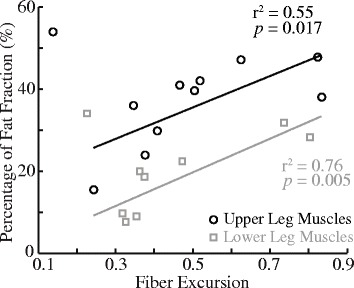
Muscle excursion was highly correlated with fat fraction for both upper leg and lower leg muscles, respectively, in patients with DMD. Fiber excursion was from Fig. 4 (red bars), and fat fraction, a metric of the extent of muscle degeneration, was measured from patients with DMD in a previous magnetic resonance imaging study (see Fig. 2 in [53]). Each circle and square represents one upper leg and lower leg muscle

Muscle fiber excursions quantified from our walking simulations were significantly correlated with the fat fractions in patients with DMD. In general, fat fractions increased with fiber excursions in walking in both upper and lower leg muscles (Fig. 7). The only exceptions were the adductor magnus in the upper leg and the peroneus in the lower leg, both of which had high fat fractions despite experiencing low fiber excursions during walking. Excluding these two muscles, linear regression analyses showed significant correlations between fiber excursions and fat fractions in upper leg (*r* ^2^ = 0.55, *p* = 0.017) and lower leg muscles (*r* ^2^ = 0.76, *p* = 0.005).

**Fig. 8:**
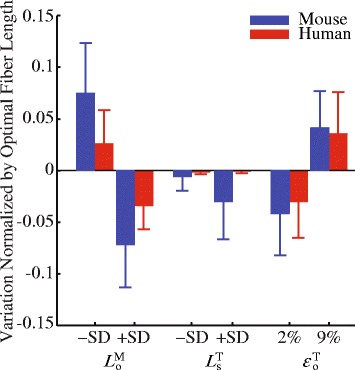
Sensitivity analyses on the parameters (mean and standard deviation) of the Hill-type muscle model, including optimal fiber length (${L}_{\mathrm{o}}^{\mathrm{M}}$), tendon slack length (${L}_{\mathrm{s}}^{\mathrm{T}}$), and tendon strain (${\varepsilon}_{\mathrm{o}}^{\mathrm{T}}$). The ${L}_{\mathrm{o}}^{\mathrm{M}}$ and ${L}_{\mathrm{s}}^{\mathrm{T}}$ values were both altered by ±1 standard deviation. The ${\varepsilon}_{\mathrm{o}}^{\mathrm{T}}$ value was altered based on plausible ranges reported in the literature from 2 to 9% (see Sensitivity analyses)

The simulation-based estimates of muscle fiber excursions during walking were not sensitive to changes of parameters in either model (Fig. 8). Varying optimal fiber length ${L}_{\mathrm{o}}^{\mathrm{M}}$ by ± 1 standard deviation resulted in the largest variation of the muscle fiber excursions. However, these variations were on average only less than $0.07\ast {L}_{\mathrm{o}}^{\mathrm{M}}$ in both the mouse hindlimb model and human lower limb model. Similarly, varying ${L}_{\mathrm{s}}^{\mathrm{T}}$ and ${\varepsilon}_{\mathrm{o}}^{\mathrm{T}}$ only led to variations of less than $0.05\ast {L}_{\mathrm{o}}^{\mathrm{M}}$ in the muscle fiber excursions.

## Discussion

The purpose of this study was to compare the muscle fiber excursions during walking of mouse hindlimb muscles with their homologs in human lower limbs. This was accomplished by running forward dynamic simulations with prescribed joint kinematics obtained from existing studies [39, 43]. Recently published musculoskeletal models of the mouse hindlimb [40] and human lower limb [37] were used to run simulations. Our simulation results suggested that most muscles (19 out of 25 muscles) in the mouse hindlimb had length changes during walking that were only about 48% of the length changes in human lower limb muscles. We found that the smaller muscle fiber excursions in mice were primarily due to differences in the walking kinematics (e.g., more crouched limb posture and reduced ranges of joint motion in mice) and muscle moment arms (relatively smaller overall in mice). We also found that the amount of fiber excursion significantly correlated to the differential muscle involvement in limb muscles in patients with DMD. These results revealed important biomechanical differences in muscle fiber function during walking, a key daily activity, and have important implications for the utility of mouse models to study neuromuscular diseases and test new treatments.

### Causes of smaller fiber excursions in mice as compared to humans

**Fig. 9:**
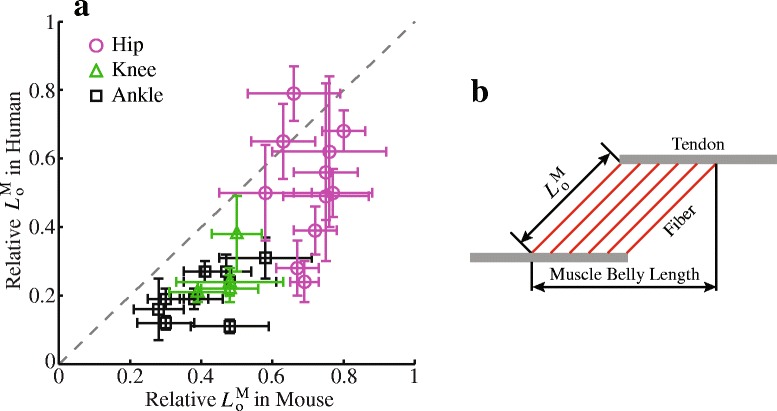
Relative optimal fiber length ${L}_{\mathrm{o}}^{\mathrm{M}}$ in mice and humans. (**a**) Comparison of relative ${L}_{\mathrm{o}}^{\mathrm{M}}$ of muscles crossing the hip, knee, and ankle joints between mice and humans. Mean and standard deviation of relative ${L}_{\mathrm{o}}^{\mathrm{M}}$ plotted in (**a**) were taken directly from Tables 3 and 4 in [41] and Table 3 in [46] for the muscles of mouse hindlimb and human lower limb, respectively. Each symbol is one muscle. The dashed line is the unity line representing the equal relative ${L}_{\mathrm{o}}^{\mathrm{M}}$ between mice and humans. (**b**) Relative ${L}_{\mathrm{o}}^{\mathrm{M}}$ was defined as ${L}_{\mathrm{o}}^{\mathrm{M}}$ normalized by muscle belly length, which was the distance from the origin of the most proximal fibers to insertion of the most distal fibers

Differences in gait kinematics, muscle geometry, and muscle architecture all contributed to the finding that muscle fiber excursions were generally smaller in mice than in humans (Fig. 4). Considering the much smaller body mass of mice vs. humans, the muscles must have adapted accordingly to meet the requirements of generating movement and supporting against gravity in these two species. Among these adaptations, muscle moment arm allometrically increases with body mass [55, 56], whereas relative optimal fiber length (optimal fiber length/muscle belly length) allometrically decreases with body mass [57], a pattern that can be observed in comparing mice with humans (Fig. 9). The combined effect was that mice had smaller normalized moment arms (moment arm/optimal fiber length) in most muscles (11 out of 15 muscles) acting across the hip and knee and all muscles acting across the ankle (Fig. 6). These geometrical and architectural adaptations in mice as well as significantly smaller ranges of joint excursion at the hip and knee (Fig. 5a) played crucial roles in the smaller fiber excursions in mice as compared with humans. Conversely, to support a larger body mass, humans have larger muscle moment arms, shorter relative optimal fiber lengths, and an upright bipedal gait, as compared to crouched, quadrupedal gait of mice [55, 56]. Although these differences provide humans with sufficient muscle strength for movement and gravitational support while preventing mechanical overload to the musculoskeletal system, they inevitably lead to greater muscle fiber excursions in humans than in mice, which may be devastating under certain neuromuscular diseases, such as DMD. In land mammals, given that normalized moment arms increase [55–57] but limb joint excursions decrease [58, 59] with increasing body mass, whether greater body mass would allometrically lead to larger limb muscle fiber excursions and therefore possibly more damaging effects in neuromuscular diseases [60] needs to be determined by more comprehensive comparative studies.

### Potential impact of fiber excursions on differential disease involvement in limb muscles

The variation in fiber excursions may contribute to the differential involvement across muscles in neuromuscular diseases. It has been well documented that in certain neuromuscular diseases, especially muscular dystrophies, muscles can be affected differently by one disease [54]. In DMD, recent imaging studies have provided a comprehensive picture of the selective muscle degeneration across lower limb muscles [53, 61]. Integrating the results from an imaging study [53], our analyses showed strong correlations between fiber excursions and fat fractions in lower limb muscle in patients with DMD (Fig. 7), which suggested a potential contribution from fiber excursion to the differential muscle involvement in DMD. In terms of *mdx* mice, to authors’ best knowledge, only a few studies have evaluated muscle degeneration across several hindlimb muscles [22, 62, 63]. It has been reported that soleus exhibits more profound degeneration as compared to extensor digitorum longus (EDL) and tibialis anterior (TA) at the beginning of ambulation (~ 3 to 4 weeks old) [62, 63]. Considering that EDL and TA of mice are more prone to damage than soleus due to their mostly fast fiber composition [20, 64], it is likely that the much greater muscle excursion in soleus than in EDL and TA (Fig. 4) plays an important role to the observed selective degeneration across these muscles. These potential links between fiber excursion and differential disease involvements within mice or humans suggested possible roles of fiber excursion on the differences in phenotypes between these two species.

### Influence of diminished fiber excursion on different phenotypes between mice and humans

The biomechanical differences in muscles during walking may contribute to different phenotypes between mouse models of neuromuscular diseases and their human counterparts, thereby impeding the translation of successful pre-clinical trials using mouse models to human trials. At comparable speed, a majority of mouse hindlimb muscles have smaller fiber excursions than those of human lower limbs (Fig. 4). These results suggested a smaller magnitude of muscle fiber lengthening (and shortening) in every gait cycle in mice compared with humans, meaning that muscles in mice hindlimbs and in human lower limbs may work under quite different repeated biomechanical loads in walking. These relatively smaller biomechanical loads in walking mice may trigger different cascades of pathological and inflammatory responses that result in differing phenotypes appearing in mouse models of neuromuscular diseases. Specifically for DMD, although several factors, such as differences in stature, growth, and inflammatory responses, may all contribute to the alleviated phenotype of *mdx* mice [1, 60], the biomechanical difference in walking revealed in this study may also play an important role. Since larger magnitudes of muscle lengthening, no matter passive or active, have been shown to cause more damage to muscles, especially dystrophic muscles [19, 65, 66], it is likely that the smaller magnitudes of muscle lengthening may alleviate muscle damage and therefore contribute to milder phenotypes in *mdx* mice hindlimb muscles compared with patients with DMD. Therefore, although *mdx* mice have been recommended as the model of choice for proof-of-concept and pre-clinical studies [67], cautious interpretation of the results is required, given the biomechanical differences during locomotion between mice and humans revealed in the current study.

### Implications for exercise protocols using mouse models

The biomechanical differences in muscles during walking may influence to what extent the current exercise protocols performed in studies using mouse models of neuromuscular disease are relevant to human patients. Exercise protocols are often used to evaluate the impact of certain types and intensities of exercises on the disease state of mouse models [68–71] and to exacerbate disease states to more rigorously evaluate potential therapeutic interventions in pre-clinical trials with mouse models [22–25]. However, it has been controversial whether the findings from these exercise studies may be extrapolated to humans due to possible biomechanical differences between mice and humans [70]. In DMD, our results showed that mice walking at the recommended exercising speed had smaller fiber excursions (Fig. 4) and therefore smaller magnitudes of muscle fiber lengthening than humans walking at comparable, natural walking speed. Thus, the treatments showing efficacy in these exercised mice may not be similarly effective in humans [26–31]. It is plausible that exercising mice at higher speeds may impose larger magnitudes of muscle fiber lengthening that is more relevant to humans [39, 49]. In addition, although alternative animal models for DMD with larger body sizes, such as golden retriever muscular dystrophy dogs [15, 72], have been considered as better models of DMD, similar studies may be needed to determine whether muscle fiber excursions in these animal models may more closely approximate those of humans in walking.

There have been studies using downhill exercise to exacerbate muscle damage in mice [73–75]. Although we have not been aware of any detailed studies on kinematics of mice walking downhill, animals with small body mass (e.g., mice) tend to use shorter strides in downhill walking than in level walking [76], which may lead to slightly smaller fiber excursions. The overall greater muscle damage in mice via downhill exercises was likely because of increased muscle activation during lengthening in order to dissipate energy and maintain locomotor velocity [76–78].

### Comparison with sarcomere length measured in experimental studies

Recent advances in imaging techniques have made possible to visualize and measure sarcomere lengths in vivo at fixed joint configurations [32, 33]. Measurements from these studies were often compared with those predicted by lumped parameter Hill-type muscle model as used in this study, which essentially assumed the whole muscle as a scaled-up sarcomere. In the human vastus lateralis and soleus, ranges of sarcomere length changes predicted by a lumped parameter model were larger than experimental measurements at two joint angles [79, 80]. Similarly, we found that the changes of sarcomere lengths of the lateral gastrocnemius and tibialis anterior muscles predicted in our simulations were relatively larger than those experimentally estimated in vivo in mice [32, 33]. The simplifications that all the fibers may be modeled by one sarcomere and have the same moment arm likely led to the overestimation of the changes in sarcomere lengths in Hill-type muscle models [81]. Finite-element models incorporating the complex details of muscle architecture [18] and/or experimental measurements of regionally heterogeneous strains in muscles [82–84] may be needed to overcome these limitations, which, however, are not practically feasible for large-scale analyses systematically across many muscles, such as this study’s analyses. Nevertheless, because the Hill-type muscle model with muscle- and species-specific architectural parameters was used to simulate fiber excursions in both mice and humans, which captures the general architectural characteristics of each individual muscle in both species (e.g., more parallel-fibered proximally and pennate distally in both species), our conclusions from comparisons of fiber excursions should not be appreciably altered.

### Sensitivity of model predictions to plausible parameter variations

Our simulated muscle fiber excursions were robust with respect to changes in model parameters within the range of physiologically plausible variations. The sensitivity analyses indicated that the predicted fiber excursion was most sensitive to optimal fiber length ${L}_{\mathrm{o}}^{\mathrm{M}}$ (Fig. 8). However, the variation in ${L}_{\mathrm{o}}^{\mathrm{M}}$ only resulted in changes of fiber excursions (<$0.07\ast {L}_{\mathrm{o}}^{\mathrm{M}}$) that were much less than average differences of muscles between mice and humans ($\left(0.25\pm 0.15\right)\ast {L}_{\mathrm{o}}^{\mathrm{M}}$). For other parameters in the Hill-type muscle model (${F}_{\mathrm{o}}^{\mathrm{M}}$, ${V}_{\mathrm{max}}^{\mathrm{M}}$, and *α*_o_), variations in ${F}_{\mathrm{o}}^{\mathrm{M}}$ do not influence the relative length changes in muscle fiber and tendon since mechanical properties of muscle fiber and tendon are both normalized to ${F}_{\mathrm{o}}^{\mathrm{M}}$ [39]. Most muscles in the mouse hindlimb and human lower limb have *α*_o_ smaller than 20° and standard deviations smaller than 5°, which should barely affect contraction dynamics of muscle-tendon units [37, 41, 45]. Although simulations of movements involving rapid muscle contractions, such as sprinting and jumping for maximum height, may be sensitive to ${V}_{\mathrm{max}}^{\mathrm{M}}$ [85, 86], simulations of walking generally require only muscle contractions at slow speed and therefore should not be sensitive to the variation in ${V}_{\mathrm{max}}^{\mathrm{M}}$ [38].

### Influences from muscle activations on fiber excursion

The influences of muscle activations on the simulated muscle fiber excursions were moderate and dependent on the ratio of tendon slack length to optimal fiber length. It is essentially impossible to acquire exact activation profiles of all 25 muscles in mouse hindlimb due to limitations with electromyography technology for measuring muscle activity in very small muscles. Therefore, fiber excursions were estimated from two sets of simulations with muscle activations at 0.05 or 1, which encompassed fiber length changes resulted from any possible muscle activation profiles during walking. On average, fully activating muscle-tendon units in our simulations moderately increased possible fiber excursions by $\left(0.05\pm 0.06\right)\ast {L}_{\mathrm{o}}^{\mathrm{M}}$ and $\left(0.09\pm 0.07\right)\ast {L}_{\mathrm{o}}^{\mathrm{M}}$ in the mouse hindlimb and human lower limb, respectively. Most muscles crossing the hip and knee joints have relatively short, stiff tendons (${L}_{\mathrm{o}}^{\mathrm{M}}/{L}_{\mathrm{s}}^{\mathrm{T}}>0.5$) that barely get stretched when these muscles are activated. Therefore, these muscles were largely not subject to influences from muscle activations [38]. In contrast, most muscles crossing the ankle joint have relatively long, compliant tendons (${L}_{\mathrm{o}}^{\mathrm{M}}/{L}_{\mathrm{s}}^{\mathrm{T}}<0.5$), in which when fully activated, tendon stretches could somewhat increase fiber excursions. Nevertheless, the influences from activation may only add limited uncertainties to the comparisons of a few out of 25 muscles (e.g., flexor digitorum longus, tibialis posterior, peroneus longus, and peroneus brevis) rather than substantially altering our overall results.

## Conclusions

The physiological and anatomical musculoskeletal structures of mice and humans are biomechanically quite different because of their very different body sizes and evolutionary histories, leading to disparate locomotor mechanisms such as limb posture and musculoskeletal stiffness [56]. To complicate the situation even more, there are biomechanical differences in how they use their musculoskeletal systems during daily locomotion as shown in the current study, and perhaps during other daily living tasks. Our simulation results demonstrated biomechanical differences that could contribute to different disease states between mouse models of neuromuscular diseases and human patients. These differences may also impede the translations of knowledge gained from mouse models to humans. By understanding and accounting for these differences, experiments in mice may be better designed to gain knowledge that could be translated to humans.

## Additional file

Additional file 1: Explanation of the rigid tendon assumption and negligible influences of the rigid tendon assumption on estimates of fiber excursion. (PDF 550 kb)

## Abbreviations

DMD: Duchenne muscular dystrophy
EDL: Extensor digitorum longus
TA: Tibialis anterior

## References

[1] Partridge TA (2013). The mdx mouse model as a surrogate for Duchenne muscular dystrophy. FEBS J.

[2] Sicinski P, Geng Y, Ryder-Cook AS, Barnard EA, Darlison MG, Barnard PJ (1989). The molecular basis of muscular dystrophy in the mdx mouse: a point mutation. Science.

[3] Gurney ME, Pu H, Chiu AY, Dal Canto MC, Polchow CY, Alexander DD, Caliendo J, Hentati A, Kwon YW, Deng HX (1994). Motor neuron degeneration in mice that express a human Cu, Zn superoxide dismutase mutation. Science.

[4] Burgess RW, Cox GA, Seburn KL (2016). Neuromuscular disease models and analysis. Methods Mol Biol.

[5] Hsieh-Li HM, Chang J-G, Jong Y-J, M-H W, Wang NM, Tsai CH, Li H (2000). A mouse model for spinal muscular atrophy. Nat Genet.

[6] Henderson VC, Kimmelman J, Fergusson D, Grimshaw JM, Hackam DG (2013). Threats to validity in the design and conduct of preclinical efficacy studies: a systematic review of guidelines for in vivo animal experiments. PLoS Med.

[7] Kornegay JN, Spurney CF, Nghiem PP, Brinkmeyer-Langford CL, Hoffman EP, Nagaraju K (2014). Pharmacologic management of Duchenne muscular dystrophy: target identification and preclinical trials. ILAR J.

[8] Benatar M (2007). Lost in translation: treatment trials in the SOD1 mouse and in human ALS. Neurobiol Dis.

[9] van der Worp HB, Howells DW, Sena ES, Porritt MJ, Rewell S, O'Collins V, Macleod MR (2010). Can animal models of disease reliably inform human studies?. PLoS Med.

[10] Ittner LM, Halliday GM, Kril JJ, Gotz J, Hodges JR, Kiernan MC (2015). FTD and ALS—translating mouse studies into clinical trials. Nat Rev Neurol.

[11] Willmann R, De Luca A, Benatar M, Grounds M, Dubach J, Raymackers JM, Nagaraju K (2012). Enhancing translation: guidelines for standard pre-clinical experiments in mdx mice. Neuromuscul Disord.

[12] Grounds MD, Radley HG, Lynch GS, Nagaraju K, De Luca A (2008). Towards developing standard operating procedures for pre-clinical testing in the mdx mouse model of Duchenne muscular dystrophy. Neurobiol Dis.

[13] Ludolph AC, Bendotti C, Blaugrund E, Chio A, Greensmith L, Loeffler JP, Mead R, Niessen HG, Petri S, Pradat PF (2010). Guidelines for preclinical animal research in ALS/MND: a consensus meeting. Amyotroph Lateral Scler.

[14] Manning J, O'Malley D (2015). What has the mdx mouse model of Duchenne muscular dystrophy contributed to our understanding of this disease?. J Muscle Res Cell Motil.

[15] McGreevy JW, Hakim CH, McIntosh MA, Duan D (2015). Animal models of Duchenne muscular dystrophy: from basic mechanisms to gene therapy. Dis Model Mech.

[16] Mah JK, Korngut L, Dykeman J, Day L, Pringsheim T, Jette N (2014). A systematic review and meta-analysis on the epidemiology of Duchenne and Becker muscular dystrophy. Neuromuscul Disord.

[17] Stedman H, Sweeney H, Shrager J, Maguire H, Panettieri R, Petrof B, Narusawa M, Leferovich J, Sladky J, Kelly A (1991). The mdx mouse diaphragm reproduces the degenerative changes of Duchenne muscular dystrophy. Nature.

[18] Hu X, Blemker SS (2015). Musculoskeletal simulation can help explain selective muscle degeneration in Duchenne muscular dystrophy. Muscle Nerve.

[19] Petrof BJ, Shrager JB, Stedman HH, Kelly AM, Sweeney HL (1993). Dystrophin protects the sarcolemma from stresses developed during muscle contraction. Proc Natl Acad Sci.

[20] Moens P, Baatsen P, Maréchal G (1993). Increased susceptibility of EDL muscles from mdx mice to damage induced by contractions with stretch. J Muscle Res Cell Motil.

[21] Use of treadmill and wheel exercise for impact on mdx mice phenotype [http://www.treat-nmd.eu/downloads/file/sops/dmd/MDX/DMD_M.2.1.003.pdf].

[22] Radley-Crabb H, Terrill J, Shavlakadze T, Tonkin J, Arthur P, Grounds M (2012). A single 30 min treadmill exercise session is suitable for ‘proof-of concept studies’ in adult mdx mice: a comparison of the early consequences of two different treadmill protocols. Neuromuscul Disord.

[23] Granchelli JA, Pollina C, Hudecki MS (2000). Pre-clinical screening of drugs using the mdx mouse. Neuromuscul Disord.

[24] De Luca A, Pierno S, Liantonio A, Cetrone M, Camerino C, Fraysse B, Mirabella M, Servidei S, Ruegg UT, Conte Camerino D (2003). Enhanced dystrophic progression in mdx mice by exercise and beneficial effects of taurine and insulin-like growth factor-1. J Pharmacol Exp Ther.

[25] Burdi R, Rolland JF, Fraysse B, Litvinova K, Cozzoli A, Giannuzzi V, Liantonio A, Camerino GM, Sblendorio V, Capogrosso RF (2009). Multiple pathological events in exercised dystrophic mdx mice are targeted by pentoxifylline: outcome of a large array of in vivo and ex vivo tests. J Appl Physiol (1985).

[26] Escolar DM, Zimmerman A, Bertorini T, Clemens PR, Connolly AM, Mesa L, Gorni K, Kornberg A, Kolski H, Kuntz N (2012). Pentoxifylline as a rescue treatment for DMD: a randomized double-blind clinical trial. Neurology.

[27] Mok E, Letellier G, Cuisset JM, Denjean A, Gottrand F, Alberti C, Hankard R (2009). Lack of functional benefit with glutamine versus placebo in Duchenne muscular dystrophy: a randomized crossover trial. PLoS One.

[28] Buyse GM, Goemans N, Henricson E, Jara A, van den Hauwe M, Leshner R, Florence JM, Mayhew JE, Escolar DM (2007). CINRG pilot trial of oxatomide in steroid-naive Duchenne muscular dystrophy. Eur J Paediatr Neurol.

[29] GSK and prosensa announce primary endpoint not met in phase III study of drisapersen in patients with Duchenne muscular dystrophy [https://globenewswire.com/news-release/2013/09/20/574726/10049265/en/GSK-and-Prosensa-Announce-Primary-Endpoint-Not-Met-in-Phase-III-Study-of-Drisapersen-in-Patients-With-Duchenne-Muscular-Dystrophy.html].

[30] Chamberlain JR, Chamberlain JS (2017). Progress toward gene therapy for Duchenne muscular dystrophy. Mol Ther.

[31] Sardone V, Zhou H, Muntoni F, Ferlini A, Falzarano MS. Antisense oligonucleotide-based therapy for neuromuscular disease. Molecules. 2017;22 10.3390/molecules22040563 PMC6154734 28379182

[32] Moo EK, Fortuna R, Sibole SC, Abusara Z, Herzog W (2016). Vivo sarcomere lengths and sarcomere elongations are not uniform across an intact muscle. Front Physiol.

[33] Llewellyn ME, Barretto RP, Delp SL, Schnitzer MJ (2008). Minimally invasive high-speed imaging of sarcomere contractile dynamics in mice and humans. Nature.

[34] Delp SL, Loan JP, Hoy MG, Zajac FE, Topp EL, Rosen JM (1990). An interactive graphics-based model of the lower extremity to study orthopaedic surgical procedures. Biomed Eng IEEE Trans.

[35] Millard M, Uchida T, Seth A, Delp SL (2013). Flexing computational muscle: modeling and simulation of musculotendon dynamics. J Biomech Eng.

[36] Arnold EM, Ward SR, Lieber RL, Delp SL (2010). A model of the lower limb for analysis of human movement. Ann Biomed Eng.

[37] Rajagopal A, Dembia CL, DeMers MS, Delp DD, Hicks JL, Delp SL (2016). Full-body musculoskeletal model for muscle-driven simulation of human gait. IEEE Trans Biomed Eng.

[38] Arnold EM, Delp SL (2011). Fibre operating lengths of human lower limb muscles during walking. Philos Trans R Soc B.

[39] Arnold EM, Hamner SR, Seth A, Millard M, Delp SL (2013). How muscle fiber lengths and velocities affect muscle force generation as humans walk and run at different speeds. J Exp Biol.

[40] Charles JP, Cappellari O, Spence AJ, Wells DJ, Hutchinson JR (2016). Muscle moment arms and sensitivity analysis of a mouse hindlimb musculoskeletal model. J Anat.

[41] Charles JP, Cappellari O, Spence AJ, Hutchinson JR, Wells DJ (2016). Musculoskeletal geometry, muscle architecture and functional specialisations of the mouse Hindlimb. PLoS One.

[42] Jenkins FA (1971). Limb posture and locomotion in the Virginia opossum (Didelphis marsupialis) and in other non-cursorial mammals. J Zool.

[43] Akay T, Tourtellotte WG, Arber S, Jessell TM (2014). Degradation of mouse locomotor pattern in the absence of proprioceptive sensory feedback. Proc Natl Acad Sci U S A.

[44] Kadaba MP, Ramakrishnan HK, Wootten ME (1990). Measurement of lower extremity kinematics during level walking. J Orthop Res.

[45] Zajac FE (1989). Muscle and tendon: properties, models, scaling, and application to biomechanics and motor control. Crit Rev Biomed Eng.

[46] Ward SR, Eng CM, Smallwood LH, Lieber RL (2009). Are current measurements of lower extremity muscle architecture accurate?. Clin Orthop Relat Res.

[47] Klein Horsman MD, Koopman HF, van der Helm FC, Prose LP, Veeger HE (2007). Morphological muscle and joint parameters for musculoskeletal modelling of the lower extremity. Clin Biomech (Bristol, Avon).

[48] Clarke KA, Still J (2001). Development and consistency of gait in the mouse. Physiol Behav.

[49] Serradj N, Jamon M (2009). The adaptation of limb kinematics to increasing walking speeds in freely moving mice 129/Sv and C57BL/6. Behav Brain Res.

[50] Delp SL, Anderson FC, Arnold AS, Loan P, Habib A, John CT, Guendelman E, Thelen DG (2007). OpenSim: open-source software to create and analyze dynamic simulations of movement. IEEE Trans Biomed Eng.

[51] Buchanan TS, Lloyd DG, Manal K, Besier TF (2004). Neuromusculoskeletal modeling: estimation of muscle forces and joint moments and movements from measurements of neural command. J Appl Biomech.

[52] An KN, Ueba Y, Chao EY, Cooney WP, Linscheid RL (1983). Tendon excursion and moment arm of index finger muscles. J Biomech.

[53] Wokke BH, van den Bergen JC, Versluis MJ, Niks EH, Milles J, Webb AG, van Zwet EW, Aartsma-Rus A, Verschuuren JJ, Kan HE (2014). Quantitative MRI and strength measurements in the assessment of muscle quality in Duchenne muscular dystrophy. Neuromuscul Disord.

[54] Emery AEH (2002). The muscular dystrophies. Lancet.

[55] Biewener AA (2005). Biomechanical consequences of scaling. J Exp Biol.

[56] Biewener AA (1990). Biomechanics of mammalian terrestrial locomotion. Science.

[57] Eng CM, Smallwood LH, Rainiero MP, Lahey M, Ward SR, Lieber RL (2008). Scaling of muscle architecture and fiber types in the rat hindlimb. J Exp Biol.

[58] Vilensky JA (1987). Locomotor behavior and control in human and non-human primates: comparisons with cats and dogs. Neurosci Biobehav Rev.

[59] McMahon TA (1975). Using body size to understand the structural design of animals: quadrupedal locomotion. J Appl Physiol.

[60] Bodor M, McDonald CM (2013). Why short stature is beneficial in Duchenne muscular dystrophy. Muscle Nerve.

[61] Li W, Zheng Y, Zhang W, Wang Z, Xiao J, Yuan Y. Progression and variation of fatty infiltration of the thigh muscles in Duchenne muscular dystrophy, a muscle magnetic resonance imaging study. Neuromuscul Disord. 2015; 10.1016/j.nmd.2015.01.003 25701397

[62] Pastoret C, Sebille A (1995). Mdx mice show progressive weakness and muscle deterioration with age. J Neurol Sci.

[63] Carnwath JW, Shotton DM (1987). Muscular dystrophy in the mdx mouse: histopathology of the soleus and extensor digitorum longus muscles. J Neurol Sci.

[64] Webster C, Silberstein L, Hays AP, Blau HM (1988). Fast muscle fibers are preferentially affected in Duchenne muscular dystrophy. Cell.

[65] Consolino CM, Brooks SV (2004). Susceptibility to sarcomere injury induced by single stretches of maximally activated muscles of mdx mice. J Appl Physiol (1985).

[66] Brooks SV, Zerba E, Faulkner JA (1995). Injury to muscle fibres after single stretches of passive and maximally stimulated muscles in mice. J Physiol.

[67] Willmann R, Possekel S, Dubach-Powell J, Meier T, Ruegg MA (2009). Mammalian animal models for Duchenne muscular dystrophy. Neuromuscul Disord.

[68] Kaczor JJ, Hall JE, Payne E, Tarnopolsky MA (2007). Low intensity training decreases markers of oxidative stress in skeletal muscle of mdx mice. Free Radic Biol Med.

[69] Veldink JH, Bar PR, Joosten EA, Otten M, Wokke JH, van den Berg LH (2003). Sexual differences in onset of disease and response to exercise in a transgenic model of ALS. Neuromuscul Disord.

[70] Carter GT, Abresch RT, Fowler Jr WM (2002). Adaptations to exercise training and contraction-induced muscle injury in animal models of muscular dystrophy. Am J Phys Med Rehabil.

[71] Kirkinezos IG, Hernandez D, Bradley WG, Moraes CT (2003). Regular exercise is beneficial to a mouse model of amyotrophic lateral sclerosis. Ann Neurol.

[72] Kornegay JN (2017). The golden retriever model of Duchenne muscular dystrophy. Skelet Muscle.

[73] Brussee V, Tardif F, Tremblay JP (1997). Muscle fibers of mdx mice are more vulnerable to exercise than those of normal mice. Neuromuscul Disord.

[74] Whitehead NP, Streamer M, Lusambili LI, Sachs F, Allen DG (2006). Streptomycin reduces stretch-induced membrane permeability in muscles from mdx mice. Neuromuscul Disord.

[75] Xu L, Park KH, Zhao L, Xu J, El Refaey M, Gao Y, Zhu H, Ma J, Han R (2016). CRISPR-mediated genome editing restores dystrophin expression and function in mdx mice. Mol Ther.

[76] Birn-Jeffery AV, Higham TE (2014). The scaling of uphill and downhill locomotion in legged animals. Integr Comp Biol.

[77] DeVita P, Helseth J, Hortobagyi T (2007). Muscles do more positive than negative work in human locomotion. J Exp Biol.

[78] Alexander N, Schwameder H (2016). Comparison of estimated and measured muscle activity during inclined walking. J Appl Biomech.

[79] Chen X, Sanchez GN, Schnitzer MJ, Delp SL (2016). Changes in sarcomere lengths of the human vastus lateralis muscle with knee flexion measured using in vivo microendoscopy. J Biomech.

[80] Chen X, Delp SL (2016). Human soleus sarcomere lengths measured using in vivo microendoscopy at two ankle flexion angles. J Biomech.

[81] Blemker SS, Delp SL (2006). Rectus femoris and vastus intermedius fiber excursions predicted by three-dimensional muscle models. J Biomech.

[82] Soman A, Hedrick TL, Biewener AA (2005). Regional patterns of pectoralis fascicle strain in the pigeon Columba livia during level flight. J Exp Biol.

[83] Camp AL, Astley HC, Horner AM, Roberts TJ, Brainerd EL (2016). Fluoromicrometry: a method for measuring muscle length dynamics with biplanar videofluoroscopy. J Exp Zool A Ecol Genet Physiol.

[84] Azizi E, Deslauriers AR (2014). Regional heterogeneity in muscle fiber strain: the role of fiber architecture. Front Physiol.

[85] Miller RH, Umberger BR, Caldwell GE (2012). Sensitivity of maximum sprinting speed to characteristic parameters of the muscle force-velocity relationship. J Biomech.

[86] Domire ZJ, Challis JH (2010). A critical examination of the maximum velocity of shortening used in simulation models of human movement. Comput Methods Biomech Biomed Engin.

