# Supplementary material for: Are mice good models for human neuromuscular disease? Comparing muscle excursions in walking between mice and humans
Source: Skelet Muscle. 2017 Nov 16;7:26. doi: 10.1186/s13395-017-0143-9 (PMC5689180; doi:10.1186/s13395-017-0143-9)
Supplement: Additional file 1: — Explanation of the rigid tendon assumption and negligible influences of the rigid tendon assumption on estimates of fiber excursion. (PDF 550 kb) [file 13395_2017_143_MOESM1_ESM.pdf]

Explanation of the rigid tendon assumption

To strike a good balance between accuracy of the simulated fiber excursions and the computational speed of simulations, some muscle-tendon units were modeled with rigid tendons (tendon strain is negligible) depending on the ratio of optimal fiber length over tendon slack length. Previous simulation studies have shown that for any muscle-tendon unit whose optimal fiber length is longer than its tendon slack length (L_o^M/L_s^T>1), tendon can be modeled as rigid to increase computational speed without affecting simulation results [1, 2]. This is because under such conditions tendon strain is negligible and the strain of the whole muscle-tendon unit is primarily taken up by the muscle fiber. The longer the optimal fiber length compared with tendon slack length is, the smaller effect that the simplification of rigid tendon has on the simulation results [2]. Therefore, in this study, to assure that the fiber excursions were estimated accurately from simulations while maintaining reasonable computational speed, tendon was modeled as rigid for muscles in which the ratio of optimal fiber length to tendon slack length of a muscle-tendon unit was bigger than two (L_o^M/L_s^T>2). Using this criterion, 19 of 44 muscle-tendon units in the mouse hindlimb model and 10 of 40 muscle-tendon units in each leg of the human lower limb model were modeled with rigid tendons.

The comparisons made in the current study included 25 muscles that have homologs in mice and humans (see Methods). For these 25 muscles in the comparisons, only 10 muscles of mice and 3 of humans in the upper leg (thigh) contained muscle-tendon units affected by the rigid tendon assumption. No lower leg muscles were modeled with rigid tendon (Fig. S1).

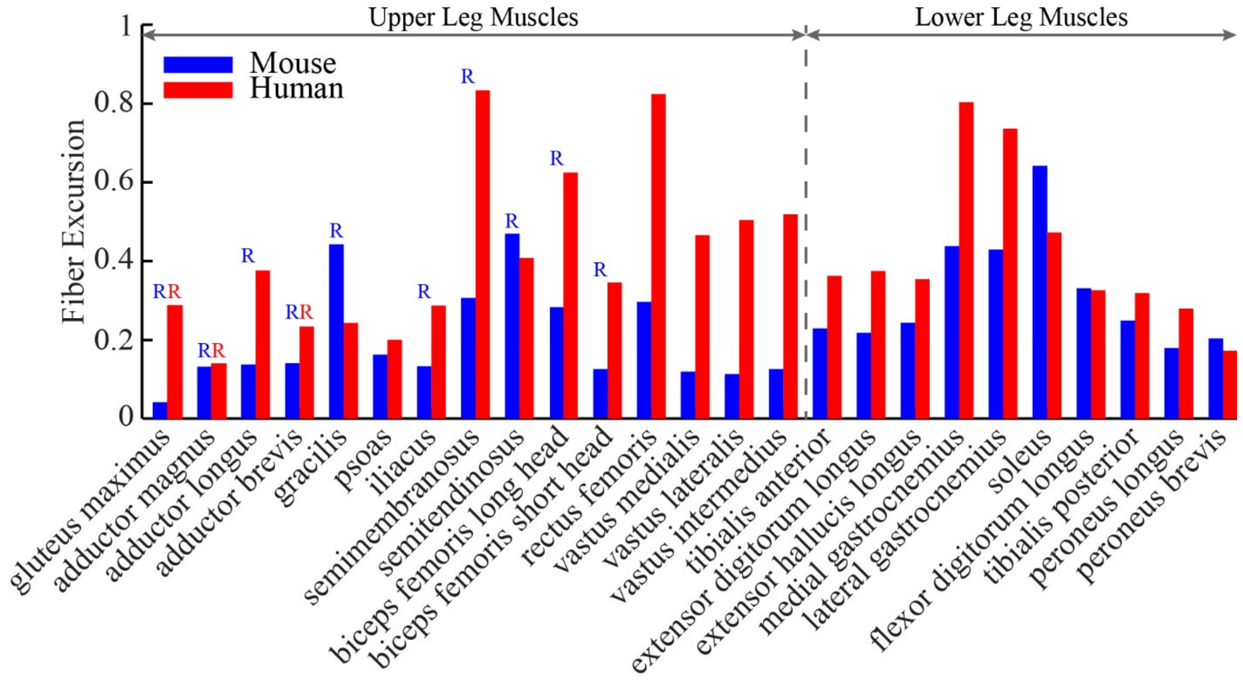

Figure S1. Ten muscles of mice and three of humans in the upper leg were affected by the rigid tendon assumption. The blue and red capital R above bars indicate the muscles that contained muscle-tendon units modeled with the rigid tendon in mice and humans, respectively.

Negligible influences of the rigid tendon assumption on estimates of fiber excursion

We compared the fiber excursions between 10 muscles modeled with rigid tendon and 15 muscles with stretchable tendon (Fig. S1), and found that there was no significant difference between the two groups (Fig. S2 A; *t*-test, $p = 0.459$). We also re-ran a set of simulations with averaged joint kinematics of mice, in which rigid tendons in 10 muscles were replaced by stretchable tendons as the remaining 15 muscles, to quantify any potential differences introduced by rigid tendon assumption. As expected, for these 10 muscles, there were negligible differences ($(0.013 \pm 0.005)*L_o^M$) in the fiber excursions between the rigid tendon and stretchable tendon (Fig. S2 B).

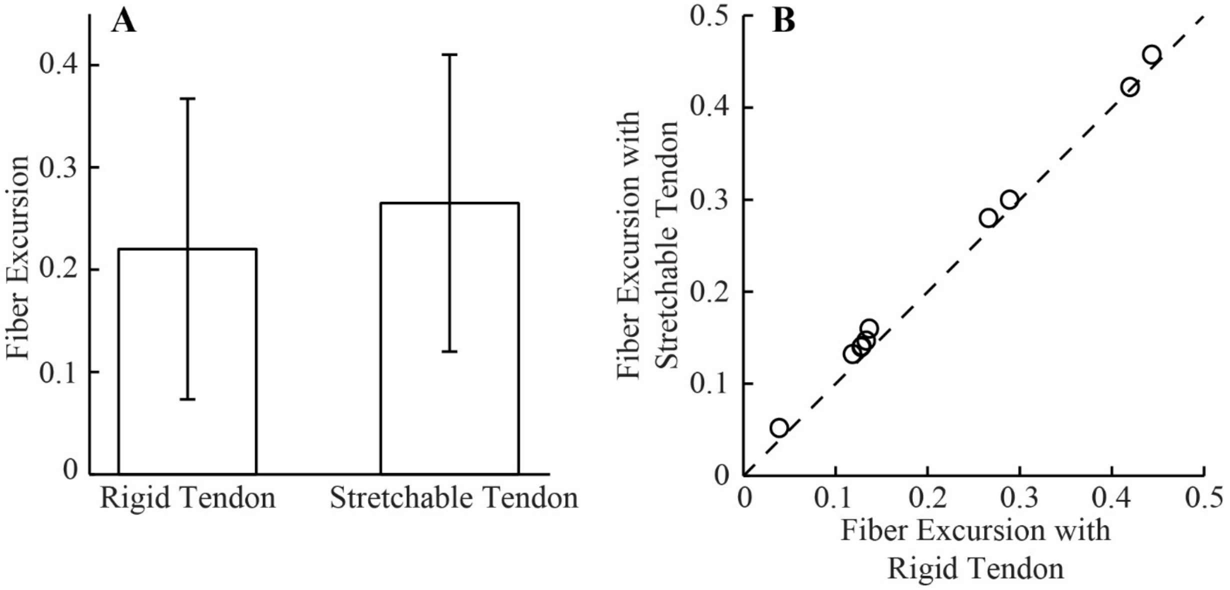

Figure S2. (A) Comparison of fiber excursions (mean and standard deviation) computed from muscles modeled with rigid tendon and stretchable tendon in mice. No significant difference was detected between the two groups. (B) Comparison of fiber excursions computed with rigid tendon and stretchable tendon in 10 upper leg muscles of mice. Across a large range of fiber excursion (roughly from $0.05*L_o^M$ to $0.45*L_o^M$), stretchable tendon only caused slightly higher fiber excursions. Each circle represents one muscle. The dashed line is the unity line representing equal fiber excursion.

References

1. Rajagopal A, Dembia CL, DeMers MS, Delp DD, Hicks JL, Delp SL: **Full-Body Musculoskeletal Model for Muscle-Driven Simulation of Human Gait.** *IEEE Trans Biomed Eng* 2016, **63:**2068-2079.
2. Millard M, Uchida T, Seth A, Delp SL: **Flexing computational muscle: modeling and simulation of musculotendon dynamics.** *J Biomech Eng* 2013, **135:**021005.
